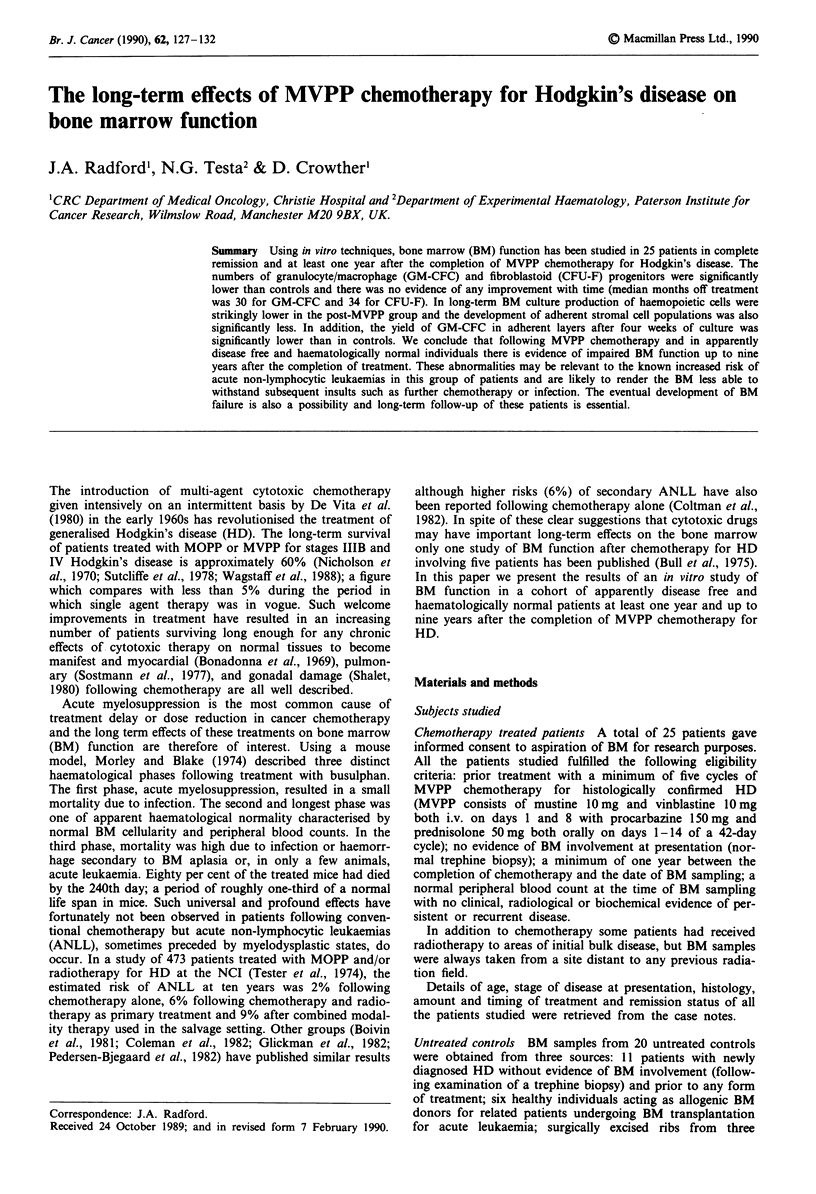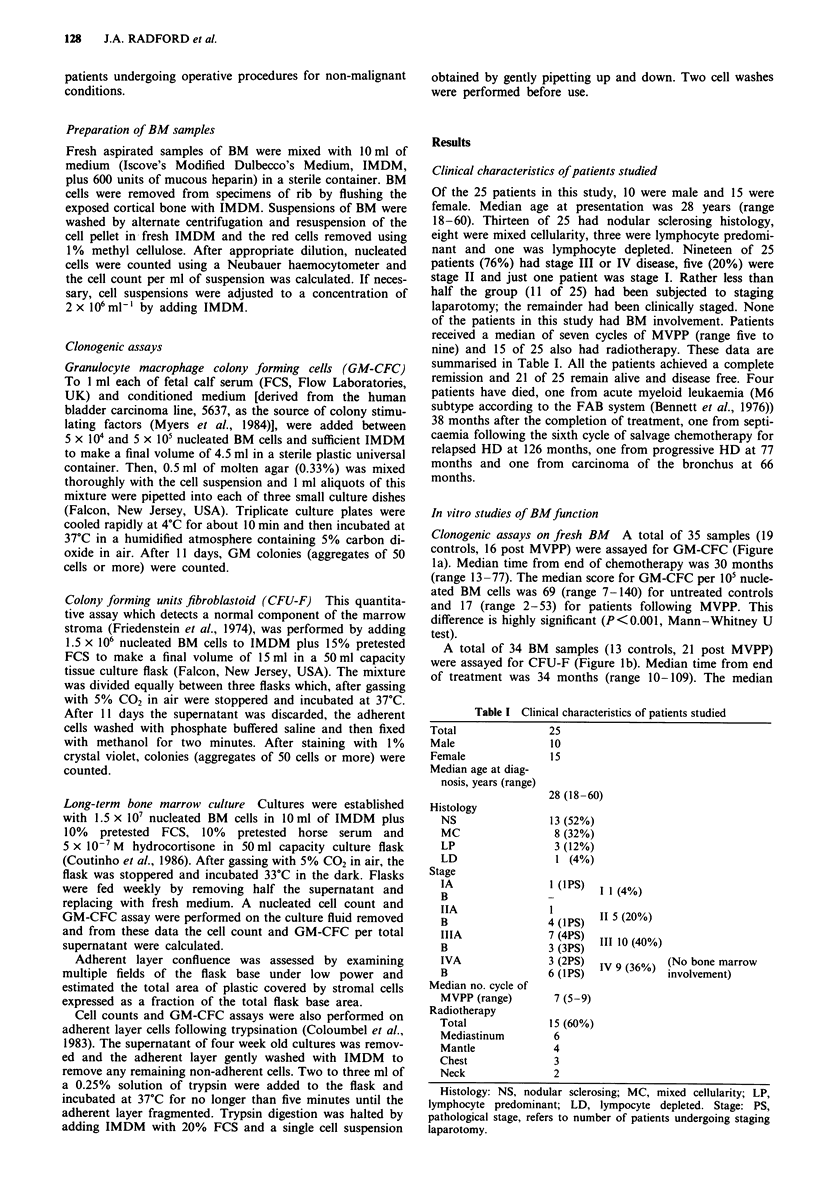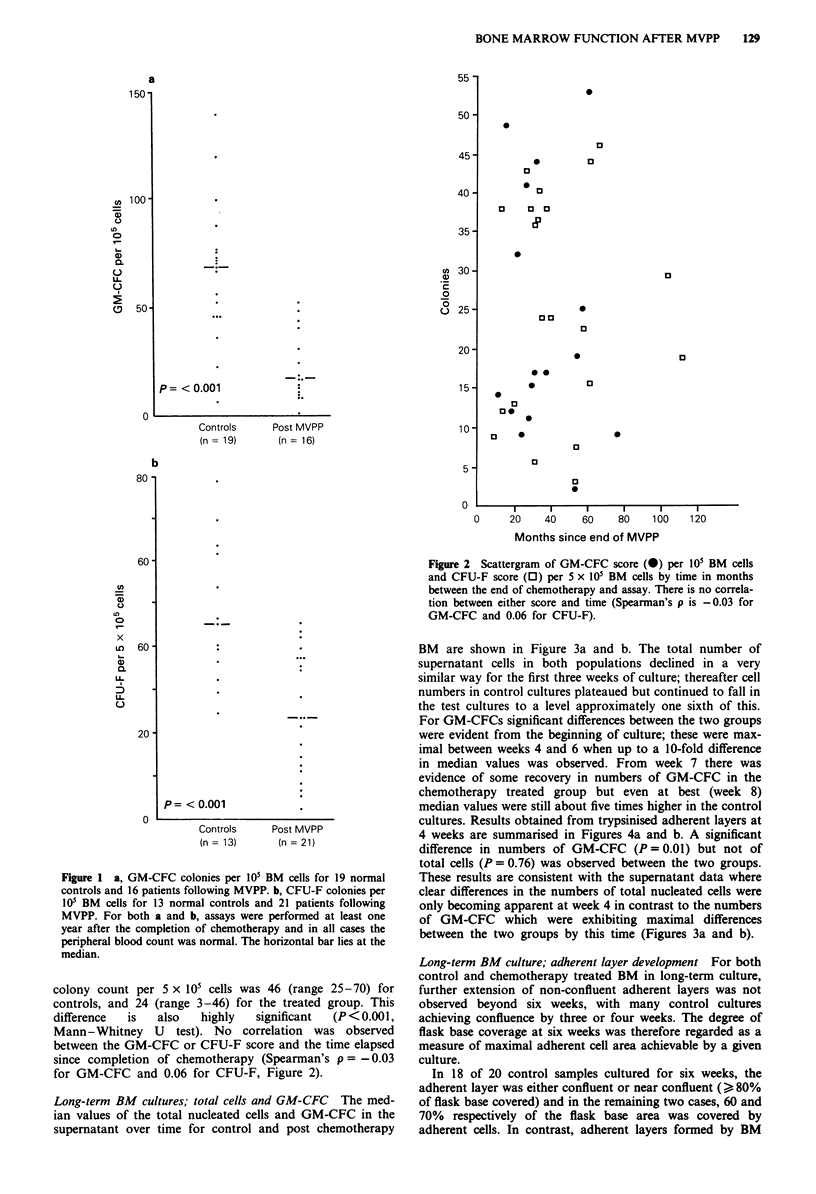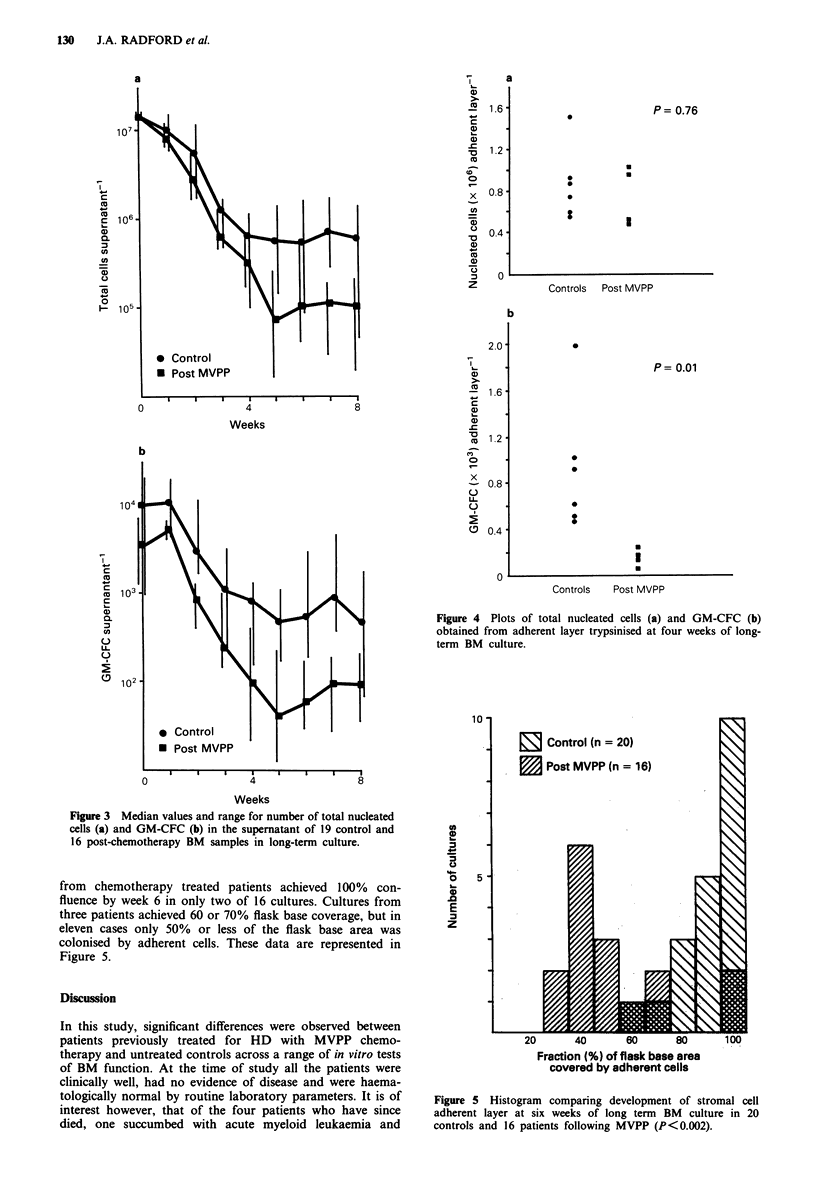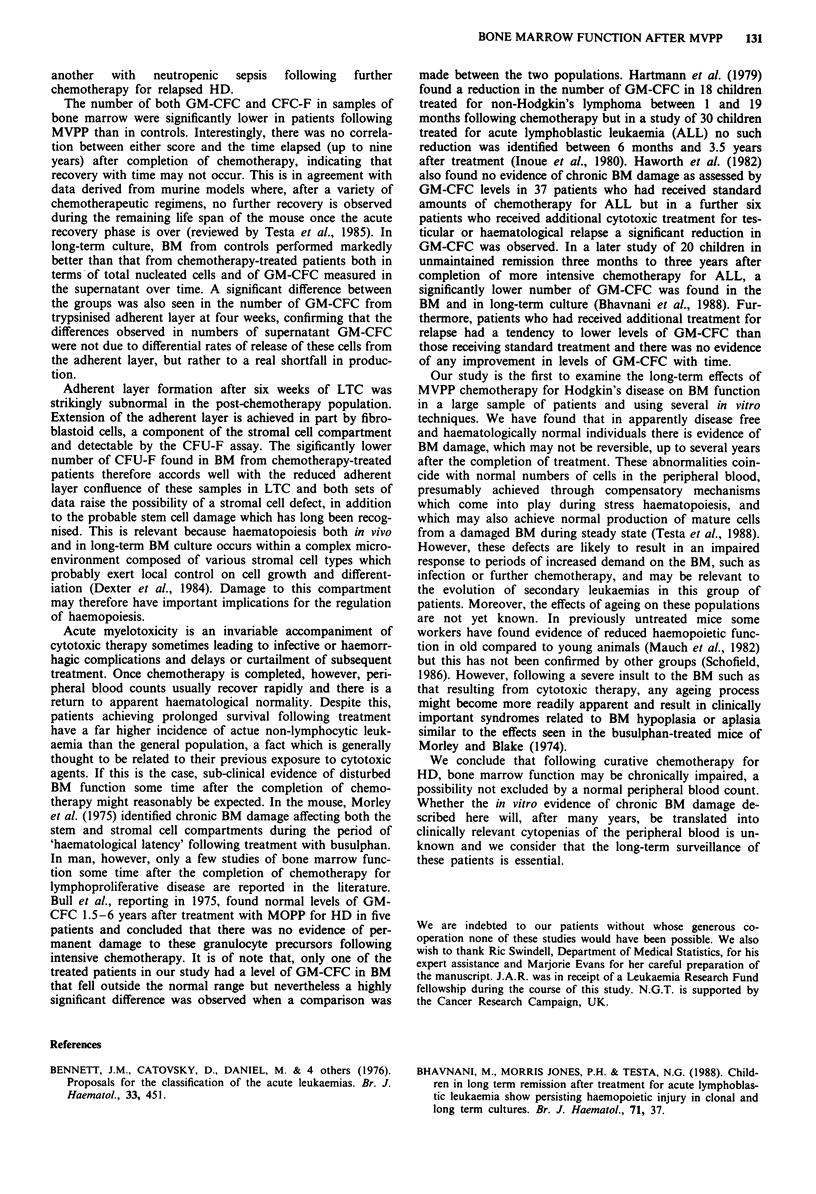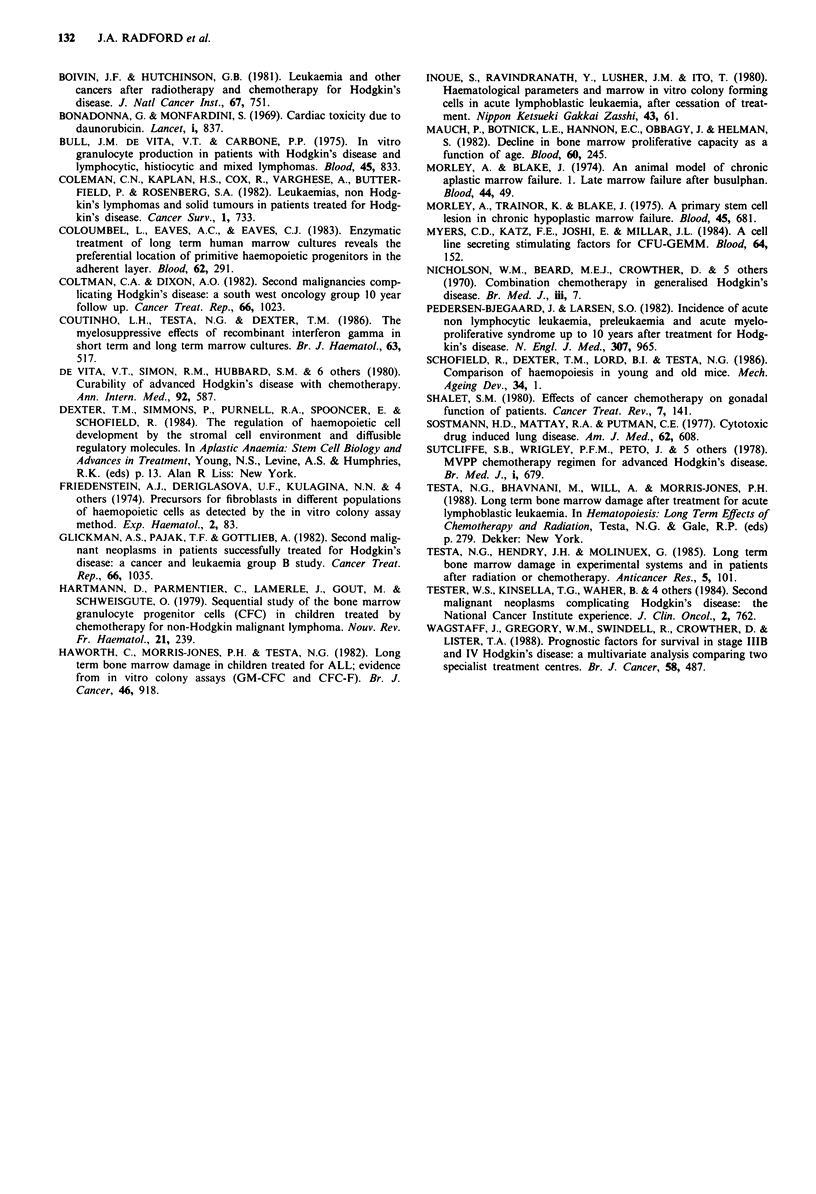# The long-term effects of MVPP chemotherapy for Hodgkin's disease on bone marrow function.

**DOI:** 10.1038/bjc.1990.243

**Published:** 1990-07

**Authors:** J. A. Radford, N. G. Testa, D. Crowther

**Affiliations:** CRC Department of Medical Oncology, Christie Hospital, Manchester, UK.

## Abstract

Using in vitro techniques, bone marrow (BM) function has been studied in 25 patients in complete remission and at least one year after the completion of MVPP chemotherapy for Hodgkin's disease. The numbers of granulocyte/macrophage (GM-CFC) and fibroblastoid (CFU-F) progenitors were significantly lower than controls and there was no evidence of any improvement with time (median months off treatment was 30 for GM-CFC and 34 for CFU-F). In long-term BM culture production of haemopoietic cells were strikingly lower in the post-MVPP group and the development of adherent stromal cell populations was also significantly less. In addition, the yield of GM-CFC in adherent layers after four weeks of culture was significantly lower than in controls. We conclude that following MVPP chemotherapy and in apparently disease free and haematologically normal individuals there is evidence of impaired BM function up to nine years after the completion of treatment. These abnormalities may be relevant to the known increased risk of acute non-lymphocytic leukaemias in this group of patients and are likely to render the BM less able to withstand subsequent insults such as further chemotherapy or infection. The eventual development of BM failure is also a possibility and long-term follow-up of these patients is essential.


 © Macmillan Press Ltd., 1990

### The long-term effects of MVPP chemotherapy for Hodgkin's disease on bone marrow function

J.A. Radford[1], N.G. Testa[2] & D. Crowther[1]

[1]*CRC Department of Medical Oncology, Christie Hospital and* [2]*Department of Experimental Haematology, Paterson Institute for Cancer Research, Wilmslow Road, Manchester M20 9BX, UK.*

**Summary** Using *in vitro* techniques, bone marrow (BM) function has been studied in 25 patients in complete remission and at least one year after the completion of MVPP chemotherapy for Hodgkin's disease. The numbers of granulocyte/macrophage (GM-CFC) and fibroblastoid (CFU-F) progenitors were significantly lower than controls and there was no evidence of any improvement with time (median months off treatment was 30 for GM-CFC and 34 for CFU-F). In long-term BM culture production of haemopoietic cells were strikingly lower in the post-MVPP group and the development of adherent stromal cell populations was also significantly less. In addition, the yield of GM-CFC in adherent layers after four weeks of culture was significantly lower than in controls. We conclude that following MVPP chemotherapy and in apparently disease free and haematologically normal individuals there is evidence of impaired BM function up to nine years after the completion of treatment. These abnormalities may be relevant to the known increased risk of acute non-lymphocytic leukaemias in this group of patients and are likely to render the BM less able to withstand subsequent insults such as further chemotherapy or infection. The eventual development of BM failure is also a possibility and long-term follow-up of these patients is essential.

The introduction of multi-agent cytotoxic chemotherapy given intensively on an intermittent basis by De Vita *et al.* (1980) in the early 1960s has revolutionised the treatment of generalised Hodgkin's disease (HD). The long-term survival of patients treated with MOPP or MVPP for stages IIIB and IV Hodgkin's disease is approximately 60% (Nicholson *et al.*, 1970; Sutcliffe *et al.*, 1978; Wagstaff *et al.*, 1988); a figure which compares with less than 5% during the period in which single agent therapy was in vogue. Such welcome improvements in treatment have resulted in an increasing number of patients surviving long enough for any chronic effects of cytotoxic therapy on normal tissues to become manifest and myocardial (Bonadonna *et al.*, 1969), pulmonary (Sostmann *et al.*, 1977), and gonadal damage (Shalet, 1980) following chemotherapy are all well described.

Acute myelosuppression is the most common cause of treatment delay or dose reduction in cancer chemotherapy and the long term effects of these treatments on bone marrow (BM) function are therefore of interest. Using a mouse model, Morley and Blake (1974) described three distinct haematological phases following treatment with busulphan. The first phase, acute myelosuppression, resulted in a small mortality due to infection. The second and longest phase was one of apparent haematological normality characterised by normal BM cellularity and peripheral blood counts. In the third phase, mortality was high due to infection or haemorrhage secondary to BM aplasia or, in only a few animals, acute leukaemia. Eighty per cent of the treated mice had died by the 240th day; a period of roughly one-third of a normal life span in mice. Such universal and profound effects have fortunately not been observed in patients following conventional chemotherapy but acute non-lymphocytic leukaemias (ANLL), sometimes preceded by myelodysplastic states, do occur. In a study of 473 patients treated with MOPP and/or radiotherapy for HD at the NCI (Tester *et al.*, 1974), the estimated risk of ANLL at ten years was 2% following chemotherapy alone, 6% following chemotherapy and radiotherapy as primary treatment and 9% after combined modality therapy used in the salvage setting. Other groups (Boivin *et al.*, 1981; Coleman *et al.*, 1982; Glickman *et al.*, 1982; Pedersen-Bjegaard *et al.*, 1982) have published similar results although higher risks (6%) of secondary ANLL have also been reported following chemotherapy alone (Coltman *et al.*, 1982). In spite of these clear suggestions that cytotoxic drugs may have important long-term effects on the bone marrow only one study of BM function after chemotherapy for HD involving five patients has been published (Bull *et al.*, 1975). In this paper we present the results of an *in vitro* study of BM function in a cohort of apparently disease free and haematologically normal patients at least one year and up to nine years after the completion of MVPP chemotherapy for HD.

Correspondence: J.A. Radford.
Received 24 October 1989; and in revised form 7 February 1990.

### Materials and methods

### Subjects studied

*Chemotherapy treated patients* A total of 25 patients gave informed consent to aspiration of BM for research purposes. All the patients studied fulfilled the following eligibility criteria: prior treatment with a minimum of five cycles of MVPP chemotherapy for histologically confirmed HD (MVPP consists of mustine 10 mg and vinblastine 10 mg both i.v. on days 1 and 8 with procarbazine 150 mg and prednisolone 50 mg both orally on days 1-14 of a 42-day cycle); no evidence of BM involvement at presentation (normal trephine biopsy); a minimum of one year between the completion of chemotherapy and the date of BM sampling; a normal peripheral blood count at the time of BM sampling with no clinical, radiological or biochemical evidence of persistent or recurrent disease.

In addition to chemotherapy some patients had received radiotherapy to areas of initial bulk disease, but BM samples were always taken from a site distant to any previous radiation field.

Details of age, stage of disease at presentation, histology, amount and timing of treatment and remission status of all the patients studied were retrieved from the case notes.

*Untreated controls* BM samples from 20 untreated controls were obtained from three sources: 11 patients with newly diagnosed HD without evidence of BM involvement (following examination of a trephine biopsy) and prior to any form of treatment; six healthy individuals acting as allogenic BM donors for related patients undergoing BM transplantation for acute leukaemia; surgically excised ribs from three patients undergoing operative procedures for non-malignant conditions.

*Preparation of BM samples*

Fresh aspirated samples of BM were mixed with 10 ml of medium (Iscove's Modified Dulbecco's Medium, IMDM, plus 600 units of mucous heparin) in a sterile container. BM cells were removed from specimens of rib by flushing the exposed cortical bone with IMDM. Suspensions of BM were washed by alternate centrifugation and resuspension of the cell pellet in fresh IMDM and the red cells removed using 1% methyl cellulose. After appropriate dilution, nucleated cells were counted using a Neubauer haemocytometer and the cell count per ml of suspension was calculated. If necessary, cell suspensions were adjusted to a concentration of $2 \times 10^6 \, ml^{-1}$ by adding IMDM.

*Clonogenic assays*

*Granulocyte macrophage colony forming cells (GM-CFC)* To 1 ml each of fetal calf serum (FCS, Flow Laboratories, UK) and conditioned medium [derived from the human bladder carcinoma line, 5637, as the source of colony stimulating factors (Myers *et al.*, 1984)], were added between $5 \times 10^4$ and $5 \times 10^5$ nucleated BM cells and sufficient IMDM to make a final volume of 4.5 ml in a sterile plastic universal container. Then, 0.5 ml of molten agar (0.33%) was mixed thoroughly with the cell suspension and 1 ml aliquots of this mixture were pipetted into each of three small culture dishes (Falcon, New Jersey, USA). Triplicate culture plates were cooled rapidly at 4°C for about 10 min and then incubated at 37°C in a humidified atmosphere containing 5% carbon dioxide in air. After 11 days, GM colonies (aggregates of 50 cells or more) were counted.

*Colony forming units fibroblastoid (CFU-F)* This quantitative assay which detects a normal component of the marrow stroma (Friedenstein *et al.*, 1974), was performed by adding $1.5 \times 10^6$ nucleated BM cells to IMDM plus 15% pretested FCS to make a final volume of 15 ml in a 50 ml capacity tissue culture flask (Falcon, New Jersey, USA). The mixture was divided equally between three flasks which, after gassing with 5% $CO_2$ in air were stoppered and incubated at 37°C. After 11 days the supernatant was discarded, the adherent cells washed with phosphate buffered saline and then fixed with methanol for two minutes. After staining with 1% crystal violet, colonies (aggregates of 50 cells or more) were counted.

*Long-term bone marrow culture* Cultures were established with $1.5 \times 10^7$ nucleated BM cells in 10 ml of IMDM plus 10% pretested FCS, 10% pretested horse serum and $5 \times 10^{-7}$ M hydrocortisone in 50 ml capacity culture flask (Coutinho *et al.*, 1986). After gassing with 5% $CO_2$ in air, the flask was stoppered and incubated 33°C in the dark. Flasks were fed weekly by removing half the supernatant and replacing with fresh medium. A nucleated cell count and GM-CFC assay were performed on the culture fluid removed and from these data the cell count and GM-CFC per total supernatant were calculated.

Adherent layer confluence was assessed by examining multiple fields of the flask base under low power and estimated the total area of plastic covered by stromal cells expressed as a fraction of the total flask base area.

Cell counts and GM-CFC assays were also performed on adherent layer cells following trypsination (Coloumbel *et al.*, 1983). The supernatant of four week old cultures was removed and the adherent layer gently washed with IMDM to remove any remaining non-adherent cells. Two to three ml of a 0.25% solution of trypsin were added to the flask and incubated at 37°C for no longer than five minutes until the adherent layer fragmented. Trypsin digestion was halted by adding IMDM with 20% FCS and a single cell suspension obtained by gently pipetting up and down. Two cell washes were performed before use.

### Results

*Clinical characteristics of patients studied*

Of the 25 patients in this study, 10 were male and 15 were female. Median age at presentation was 28 years (range 18–60). Thirteen of 25 had nodular sclerosing histology, eight were mixed cellularity, three were lymphocyte predominant and one was lymphocyte depleted. Nineteen of 25 patients (76%) had stage III or IV disease, five (20%) were stage II and just one patient was stage I. Rather less than half the group (11 of 25) had been subjected to staging laparotomy; the remainder had been clinically staged. None of the patients in this study had BM involvement. Patients received a median of seven cycles of MVPP (range five to nine) and 15 of 25 also had radiotherapy. These data are summarised in Table I. All the patients achieved a complete remission and 21 of 25 remain alive and disease free. Four patients have died, one from acute myeloid leukaemia (M6 subtype according to the FAB system (Bennett *et al.*, 1976)) 38 months after the completion of treatment, one from septicaemia following the sixth cycle of salvage chemotherapy for relapsed HD at 126 months, one from progressive HD at 77 months and one from carcinoma of the bronchus at 66 months.

*In vitro studies of BM function*

*Clonogenic assays on fresh BM* A total of 35 samples (19 controls, 16 post MVPP) were assayed for GM-CFC (Figure 1a). Median time from end of chemotherapy was 30 months (range 13–77). The median score for GM-CFC per $10^5$ nucleated BM cells was 69 (range 7–140) for untreated controls and 17 (range 2–53) for patients following MVPP. This difference is highly significant ($P<0.001$, Mann–Whitney U test).

A total of 34 BM samples (13 controls, 21 post MVPP) were assayed for CFU-F (Figure 1b). Median time from end of treatment was 34 months (range 10–109). The median

**Table I** Clinical characteristics of patients studied

| | | | |
|---|---|---|---|
| Total | 25 | | |
| Male | 10 | | |
| Female | 15 | | |
| Median age at diagnosis, years (range) | 28 (18–60) | | |
| Histology | | | |
| NS | 13 (52%) | | |
| MC | 8 (32%) | | |
| LP | 3 (12%) | | |
| LD | 1 (4%) | | |
| Stage | | | |
| IA | 1 (1PS) | I 1 (4%) | |
| B | – | | |
| IIA | 1 | II 5 (20%) | |
| B | 4 (1PS) | | |
| IIIA | 7 (4PS) | III 10 (40%) | |
| B | 3 (3PS) | | |
| IVA | 3 (2PS) | IV 9 (36%) | (No bone marrow involvement) |
| B | 6 (1PS) | | |
| Median no. cycle of MVPP (range) | 7 (5–9) | | |
| Radiotherapy | | | |
| Total | 15 (60%) | | |
| Mediastinum | 6 | | |
| Mantle | 4 | | |
| Chest | 3 | | |
| Neck | 2 | | |

Histology: NS, nodular sclerosing; MC, mixed cellularity; LP, lymphocyte predominant; LD, lympocyte depleted. Stage: PS, pathological stage, refers to number of patients undergoing staging laparotomy.

**Figure 1** **a**, GM-CFC colonies per $10^5$ BM cells for 19 normal controls and 16 patients following MVPP. **b**, CFU-F colonies per $10^5$ BM cells for 13 normal controls and 21 patients following MVPP. For both **a** and **b**, assays were performed at least one year after the completion of chemotherapy and in all cases the peripheral blood count was normal. The horizontal bar lies at the median.

colony count per $5 \times 10^5$ cells was 46 (range 25–70) for controls, and 24 (range 3–46) for the treated group. This difference is also highly significant ($P < 0.001$, Mann–Whitney U test). No correlation was observed between the GM-CFC or CFU-F score and the time elapsed since completion of chemotherapy (Spearman's $\rho = -0.03$ for GM-CFC and 0.06 for CFU-F, Figure 2).

*Long-term BM cultures; total cells and GM-CFC* The median values of the total nucleated cells and GM-CFC in the supernatant over time for control and post chemotherapy

**Figure 2** Scattergram of GM-CFC score (●) per $10^5$ BM cells and CFU-F score (□) per $5 \times 10^5$ BM cells by time in months between the end of chemotherapy and assay. There is no correlation between either score and time (Spearman's $\rho$ is $-0.03$ for GM-CFC and 0.06 for CFU-F).

BM are shown in Figure 3a and b. The total number of supernatant cells in both populations declined in a very similar way for the first three weeks of culture; thereafter cell numbers in control cultures plateaued but continued to fall in the test cultures to a level approximately one sixth of this. For GM-CFCs significant differences between the two groups were evident from the beginning of culture; these were maximal between weeks 4 and 6 when up to a 10-fold difference in median values was observed. From week 7 there was evidence of some recovery in numbers of GM-CFC in the chemotherapy treated group but even at best (week 8) median values were still about five times higher in the control cultures. Results obtained from trypsinised adherent layers at 4 weeks are summarised in Figures 4a and b. A significant difference in numbers of GM-CFC ($P = 0.01$) but not of total cells ($P = 0.76$) was observed between the two groups. These results are consistent with the supernatant data where clear differences in the numbers of total nucleated cells were only becoming apparent at week 4 in contrast to the numbers of GM-CFC which were exhibiting maximal differences between the two groups by this time (Figures 3a and b).

*Long-term BM culture; adherent layer development* For both control and chemotherapy treated BM in long-term culture, further extension of non-confluent adherent layers was not observed beyond six weeks, with many control cultures achieving confluence by three or four weeks. The degree of flask base coverage at six weeks was therefore regarded as a measure of maximal adherent cell area achievable by a given culture.

In 18 of 20 control samples cultured for six weeks, the adherent layer was either confluent or near confluent ($\geqslant 80\%$ of flask base covered) and in the remaining two cases, 60 and 70% respectively of the flask base area was covered by adherent cells. In contrast, adherent layers formed by BM

**Figure 3** Median values and range for number of total nucleated cells (**a**) and GM-CFC (**b**) in the supernatant of 19 control and 16 post-chemotherapy BM samples in long-term culture.

from chemotherapy treated patients achieved 100% confluence by week 6 in only two of 16 cultures. Cultures from three patients achieved 60 or 70% flask base coverage, but in eleven cases only 50% or less of the flask base area was colonised by adherent cells. These data are represented in Figure 5.

### Discussion

In this study, significant differences were observed between patients previously treated for HD with MVPP chemotherapy and untreated controls across a range of *in vitro* tests of BM function. At the time of study all the patients were clinically well, had no evidence of disease and were haematologically normal by routine laboratory parameters. It is of interest however, that of the four patients who have since died, one succumbed with acute myeloid leukaemia and

**Figure 4** Plots of total nucleated cells (**a**) and GM-CFC (**b**) obtained from adherent layer trypsinised at four weeks of long-term BM culture.

**Figure 5** Histogram comparing development of stromal cell adherent layer at six weeks of long term BM culture in 20 controls and 16 patients following MVPP ($P < 0.002$).

another with neutropenic sepsis following further chemotherapy for relapsed HD.

The number of both GM-CFC and CFC-F in samples of bone marrow were significantly lower in patients following MVPP than in controls. Interestingly, there was no correlation between either score and the time elapsed (up to nine years) after completion of chemotherapy, indicating that recovery with time may not occur. This is in agreement with data derived from murine models where, after a variety of chemotherapeutic regimens, no further recovery is observed during the remaining life span of the mouse once the acute recovery phase is over (reviewed by Testa *et al.*, 1985). In long-term culture, BM from controls performed markedly better than that from chemotherapy-treated patients both in terms of total nucleated cells and of GM-CFC measured in the supernatant over time. A significant difference between the groups was also seen in the number of GM-CFC from trypsinised adherent layer at four weeks, confirming that the differences observed in numbers of supernatant GM-CFC were not due to differential rates of release of these cells from the adherent layer, but rather to a real shortfall in production.

Adherent layer formation after six weeks of LTC was strikingly subnormal in the post-chemotherapy population. Extension of the adherent layer is achieved in part by fibroblastoid cells, a component of the stromal cell compartment and detectable by the CFU-F assay. The sigificantly lower number of CFU-F found in BM from chemotherapy-treated patients therefore accords well with the reduced adherent layer confluence of these samples in LTC and both sets of data raise the possibility of a stromal cell defect, in addition to the probable stem cell damage which has long been recognised. This is relevant because haematopoiesis both *in vivo* and in long-term BM culture occurs within a complex microenvironment composed of various stromal cell types which probably exert local control on cell growth and differentiation (Dexter *et al.*, 1984). Damage to this compartment may therefore have important implications for the regulation of haemopoiesis.

Acute myelotoxicity is an invariable accompaniment of cytotoxic therapy sometimes leading to infective or haemorrhagic complications and delays or curtailment of subsequent treatment. Once chemotherapy is completed, however, peripheral blood counts usually recover rapidly and there is a return to apparent haematological normality. Despite this, patients achieving prolonged survival following treatment have a far higher incidence of actue non-lymphocytic leukaemia than the general population, a fact which is generally thought to be related to their previous exposure to cytotoxic agents. If this is the case, sub-clinical evidence of disturbed BM function some time after the completion of chemotherapy might reasonably be expected. In the mouse, Morley *et al.* (1975) identified chronic BM damage affecting both the stem and stromal cell compartments during the period of 'haematological latency' following treatment with busulphan. In man, however, only a few studies of bone marrow function some time after the completion of chemotherapy for lymphoproliferative disease are reported in the literature. Bull *et al.*, reporting in 1975, found normal levels of GM-CFC 1.5–6 years after treatment with MOPP for HD in five patients and concluded that there was no evidence of permanent damage to these granulocyte precursors following intensive chemotherapy. It is of note that, only one of the treated patients in our study had a level of GM-CFC in BM that fell outside the normal range but nevertheless a highly significant difference was observed when a comparison was made between the two populations. Hartmann *et al.* (1979) found a reduction in the number of GM-CFC in 18 children treated for non-Hodgkin's lymphoma between 1 and 19 months following chemotherapy but in a study of 30 children treated for acute lymphoblastic leukaemia (ALL) no such reduction was identified between 6 months and 3.5 years after treatment (Inoue *et al.*, 1980). Haworth *et al.* (1982) also found no evidence of chronic BM damage as assessed by GM-CFC levels in 37 patients who had received standard amounts of chemotherapy for ALL but in a further six patients who received additional cytotoxic treatment for testicular or haematological relapse a significant reduction in GM-CFC was observed. In a later study of 20 children in unmaintained remission three months to three years after completion of more intensive chemotherapy for ALL, a significantly lower number of GM-CFC was found in the BM and in long-term culture (Bhavnani *et al.*, 1988). Furthermore, patients who had received additional treatment for relapse had a tendency to lower levels of GM-CFC than those receiving standard treatment and there was no evidence of any improvement in levels of GM-CFC with time.

Our study is the first to examine the long-term effects of MVPP chemotherapy for Hodgkin's disease on BM function in a large sample of patients and using several *in vitro* techniques. We have found that in apparently disease free and haematologically normal individuals there is evidence of BM damage, which may not be reversible, up to several years after the completion of treatment. These abnormalities coincide with normal numbers of cells in the peripheral blood, presumably achieved through compensatory mechanisms which come into play during stress haematopoiesis, and which may also achieve normal production of mature cells from a damaged BM during steady state (Testa *et al.*, 1988). However, these defects are likely to result in an impaired response to periods of increased demand on the BM, such as infection or further chemotherapy, and may be relevant to the evolution of secondary leukaemias in this group of patients. Moreover, the effects of ageing on these populations are not yet known. In previously untreated mice some workers have found evidence of reduced haemopoietic function in old compared to young animals (Mauch *et al.*, 1982) but this has not been confirmed by other groups (Schofield, 1986). However, following a severe insult to the BM such as that resulting from cytotoxic therapy, any ageing process might become more readily apparent and result in clinically important syndromes related to BM hypoplasia or aplasia similar to the effects seen in the busulphan-treated mice of Morley and Blake (1974).

We conclude that following curative chemotherapy for HD, bone marrow function may be chronically impaired, a possibility not excluded by a normal peripheral blood count. Whether the *in vitro* evidence of chronic BM damage described here will, after many years, be translated into clinically relevant cytopenias of the peripheral blood is unknown and we consider that the long-term surveillance of these patients is essential.

We are indebted to our patients without whose generous cooperation none of these studies would have been possible. We also wish to thank Ric Swindell, Department of Medical Statistics, for his expert assistance and Marjorie Evans for her careful preparation of the manuscript. J.A.R. was in receipt of a Leukaemia Research Fund fellowship during the course of this study. N.G.T. is supported by the Cancer Research Campaign, UK.

### References

BENNETT, J.M., CATOVSKY, D., DANIEL, M. & 4 others (1976). Proposals for the classification of the acute leukaemias. *Br. J. Haematol.*, **33**, 451.

BHAVNANI, M., MORRIS JONES, P.H. & TESTA, N.G. (1988). Children in long term remission after treatment for acute lymphoblastic leukaemia show persisting haemopoietic injury in clonal and long term cultures. *Br. J. Haematol.*, **71**, 37.

BOIVIN, J.F. & HUTCHINSON, G.B. (1981). Leukaemia and other cancers after radiotherapy and chemotherapy for Hodgkin's disease. *J. Natl Cancer Inst.*, **67**, 751.

BONADONNA, G. & MONFARDINI, S. (1969). Cardiac toxicity due to daunorubicin. *Lancet*, **i**, 837.

BULL, J.M. DE VITA, V.T. & CARBONE, P.P. (1975). In vitro granulocyte production in patients with Hodgkin's disease and lymphocytic, histiocytic and mixed lymphomas. *Blood*, **45**, 833.

COLEMAN, C.N., KAPLAN, H.S., COX, R., VARGHESE, A., BUTTERFIELD, P. & ROSENBERG, S.A. (1982). Leukaemias, non Hodgkin's lymphomas and solid tumours in patients treated for Hodgkin's disease. *Cancer Surv.*, **1**, 733.

COLOUMBEL, L., EAVES, A.C., & EAVES, C.J. (1983). Enzymatic treatment of long term human marrow cultures reveals the preferential location of primitive haemopoietic progenitors in the adherent layer. *Blood*, **62**, 291.

COLTMAN, C.A. & DIXON, A.O. (1982). Second malignancies complicating Hodgkin's disease: a south west oncology group 10 year follow up. *Cancer Treat. Rep.*, **66**, 1023.

COUTINHO, L.H., TESTA, N.G. & DEXTER, T.M. (1986). The myelosuppressive effects of recombinant interferon gamma in short term and long term marrow cultures. *Br. J. Haematol.*, **63**, 517.

DE VITA, V.T., SIMON, R.M., HUBBARD, S.M. & 6 others (1980). Curability of advanced Hodgkin's disease with chemotherapy. *Ann. Intern. Med.*, **92**, 587.

DEXTER, T.M., SIMMONS, P., PURNELL, R.A., SPOONCER, E. & SCHOFIELD, R. (1984). The regulation of haemopoietic cell development by the stromal cell environment and diffusible regulatory molecules. In *Aplastic Anaemia: Stem Cell Biology and Advances in Treatment*, Young, N.S., Levine, A.S. & Humphries, R.K. (eds) p. 13. Alan R Liss: New York.

FRIEDENSTEIN, A.J., DERIGLASOVA, U.F., KULAGINA, N.N. & 4 others (1974). Precursors for fibroblasts in different populations of haemopoietic cells as detected by the in vitro colony assay method. *Exp. Haematol.*, **2**, 83.

GLICKMAN, A.S., PAJAK, T.F. & GOTTLIEB, A. (1982). Second malignant neoplasms in patients successfully treated for Hodgkin's disease: a cancer and leukaemia group B study. *Cancer Treat. Rep.*, **66**, 1035.

HARTMANN, D., PARMENTIER, C., LAMERLE, J., GOUT, M. & SCHWEISGUTE, O. (1979). Sequential study of the bone marrow granulocyte progenitor cells (CFC) in children treated by chemotherapy for non-Hodgkin malignant lymphoma. *Nouv. Rev. Fr. Haematol.*, **21**, 239.

HAWORTH, C., MORRIS-JONES, P.H. & TESTA, N.G. (1982). Long term bone marrow damage in children treated for ALL; evidence from in vitro colony assays (GM-CFC and CFC-F). *Br. J. Cancer*, **46**, 918.

INOUE, S., RAVINDRANATH, Y., LUSHER, J.M. & ITO, T. (1980). Haematological parameters and marrow in vitro colony forming cells in acute lymphoblastic leukaemia, after cessation of treatment. *Nippon Ketsueki Gakkai Zasshi*, **43**, 61.

MAUCH, P., BOTNICK, L.E., HANNON, E.C., OBBAGY, J. & HELMAN, S. (1982). Decline in bone marrow proliferative capacity as a function of age. *Blood*, **60**, 245.

MORLEY, A. & BLAKE, J. (1974). An animal model of chronic aplastic marrow failure. 1. Late marrow failure after busulphan. *Blood*, **44**, 49.

MORLEY, A., TRAINOR, K. & BLAKE, J. (1975). A primary stem cell lesion in chronic hypoplastic marrow failure. *Blood*, **45**, 681.

MYERS, C.D., KATZ, F.E., JOSHI, E. & MILLAR, J.L. (1984). A cell line secreting stimulating factors for CFU-GEMM. *Blood*, **64**, 152.

NICHOLSON, W.M., BEARD, M.E.J., CROWTHER, D. & 5 others (1970). Combination chemotherapy in generalised Hodgkin's disease. *Br. Med. J.*, **iii**, 7.

PEDERSEN-BJEGAARD, J. & LARSEN, S.O. (1982). Incidence of acute non lymphocytic leukaemia, preleukaemia and acute myeloproliferative syndrome up to 10 years after treatment for Hodgkin's disease. *N. Engl. J. Med.*, **307**, 965.

SCHOFIELD, R., DEXTER, T.M., LORD, B.I. & TESTA, N.G. (1986). Comparison of haemopoiesis in young and old mice. *Mech. Ageing Dev.*, **34**, 1.

SHALET, S.M. (1980). Effects of cancer chemotherapy on gonadal function of patients. *Cancer Treat. Rev.*, **7**, 141.

SOSTMANN, H.D., MATTAY, R.A. & PUTMAN, C.E. (1977). Cytotoxic drug induced lung disease. *Am. J. Med.*, **62**, 608.

SUTCLIFFE, S.B., WRIGLEY, P.F.M., PETO, J. & 5 others (1978). MVPP chemotherapy regimen for advanced Hodgkin's disease. *Br. Med. J.*, **i**, 679.

TESTA, N.G., BHAVNANI, M., WILL, A. & MORRIS-JONES, P.H. (1988). Long term bone marrow damage after treatment for acute lymphoblastic leukaemia. In *Hematopoiesis: Long Term Effects of Chemotherapy and Radiation*, Testa, N.G. & Gale, R.P. (eds) p. 279. Dekker: New York.

TESTA, N.G., HENDRY, J.H. & MOLINUEX, G. (1985). Long term bone marrow damage in experimental systems and in patients after radiation or chemotherapy. *Anticancer Res.*, **5**, 101.

TESTER, W.S., KINSELLA, T.G., WAHER, B. & 4 others (1984). Second malignant neoplasms complicating Hodgkin's disease: the National Cancer Institute experience. *J. Clin. Oncol.*, **2**, 762.

WAGSTAFF, J., GREGORY, W.M., SWINDELL, R., CROWTHER, D. & LISTER, T.A. (1988). Prognostic factors for survival in stage IIIB and IV Hodgkin's disease: a multivariate analysis comparing two specialist treatment centres. *Br. J. Cancer*, **58**, 487.